# Impact of Estimated Plasma Volume Status on Mortality in Right Heart Failure Patients: A Retrospective Cohort Study in Indonesia

**DOI:** 10.5334/gh.1136

**Published:** 2022-08-25

**Authors:** Hannah Soetjoadi, Dewi Friska, Bambang Budi Siswanto, Hary Sakti Muliawan

**Affiliations:** 1 Faculty of Medicine, Universitas Indonesia, Jakarta, Indonesia; 2 Department of Cardiology and Vascular Medicine, Faculty of Medicine, Universitas Indonesia, Jakarta, Indonesia

**Keywords:** plasma volume, mortality, prognosis, right ventricular failure

## Abstract

**Background:**

Plasma volume (PV) expansion hallmarks the syndrome of heart failure (HF) but is difficult to be quantified noninvasively. Estimated plasma volume status (ePVS) has marked prognostic utility in the failing left heart, however its use in right heart failure (RHF) remains unknown. This study aims to investigate the prognostic value of ePVS among isolated RHF patients.

**Methods:**

We retrospectively collected 208 patients admitted for RHF in our hospital from the electronic database from 2017 to 2019. ePVS was calculated using the Hakim formula. Patients were divided into low and high groups based on their PV value. Logistic regression was used to compare the odds of in-hospital mortality between these groups.

**Results:**

The overall in-hospital mortality was 12.5%, tripled from the low group to the high group (6.7% vs. 18.3%), within a median of 6 (3–19) days. High ePVS significantly predicted mortality in RHF, even after being adjusted for demographic, hemodynamic, chemistry, and medication variables (adjusted OR: 5.83, 95% CI: 1.62–20.95, *p* < 0.01).

**Conclusion:**

ePVS is associated with in-hospital mortality among isolated RHF patients. Given not only the wide accessibility of hemogram but also the low cost and the rapid quantification of relative PV, this simple tool can potentially aid in optimizing RHF management, especially in rural area, although further evaluation is warranted.

## Background

Heart failure (HF) has been globally known to put a significant burden on patients and the healthcare system [1] in Indonesia without exception. It is estimated that 530,000 (0.3%) Indonesian citizens were diagnosed or experienced the symptoms of HF [2], and approximately 17.2% of them died in hospital [3]. The main reason for urgent HF hospitalization is congestion, which indicates a worsening condition. Because acute HF patients who are admitted with features of fluid overload and are discharged with residual congestion have higher rates of mortality and hospital readmissions [4], the assessment of plasma volume (PV) excess has been put forward. It aids in detecting volume expansion and relieving congestion even before any signs and symptoms are overt. However, the established methods to measure PV are limited to invasive and expensive means [5,6]. Hence, managing HF in low- and middle-income countries like Indonesia still poses some challenges due to the lack of resource and access.

In light of these difficulties, various alternative methods have been developed. A formula derived from body weight and hematocrit has been widely used to estimates PV status (ePVS), the degree of deviation from patient’s ideal PV [7]. The result has been repeatedly reported to predict the clinical outcomes in patients with left-sided HF (LHF). Increased ePVS, which indicates a greater degree of volume expansion and congestion, is associated with a higher risk mortality and rehospitalization among acute [8,9,10] HF patients with reduced [8,9] and preserved [10] ejection fraction. Despite its good prognostic value, calculated PV only modestly reflects the measured “true” PV [7,10] and thus appears insufficient to be incorporated to HF guidelines. In the setting of rural area with limited medical facilities, however, efforts to improve HF management are necessary, and this equation can still play its role with caution in mind.

Although PV mainly assesses the systemic component of congestion [11], it has not been explored in the right HF (RHF) population who mainly present with extrapulmonary congestion at admission. Therefore, this study aims to investigate the impact of ePVS on mortality among patients with acute RHF. Using readily available clinical data, ePVS is quick, is cheap, needs no special training, and evaluates PV expansion, which facilitates for optimizing management of RHF.

## Method

### Subjects

The study protocol was approved by the Research Ethics Committee at the National Cardiovascular Center Harapan Kita. Following the guidelines provided, written informed consent was waived because of the retrospective study design. Of 550 data obtained, this study collected 208 data of isolated RHF patients hospitalized at National Cardiovascular Center Harapan Kita, Jakarta, Indonesia, from 2017 to 2019. The diagnosis was filtered using the keywords “RHF” and “right” from the electronic database of HF patients. It was then manually evaluated by looking at the clinical history and physical and supporting examinations. Echocardiography results were particularly relied on for establishing the diagnosis of RHF: right atrium (RA) enlargement, right ventricle (RV) dilatation, or tricuspid annular plane systolic excursion (TAPSE) <15% [12]. In contrast, the exclusion criteria were patients aged below 18 years old or who had a missing data on body weight and/or hematocrit. Those who had a major involvement of left heart disease (left ventricular ejection fraction [LVEF] ≤40%, mitral regurgitation, mitral stenosis, or diastolic dysfunction [13]) or noncardiac disease were also excluded.

### Data Collection

**Figure 1:**
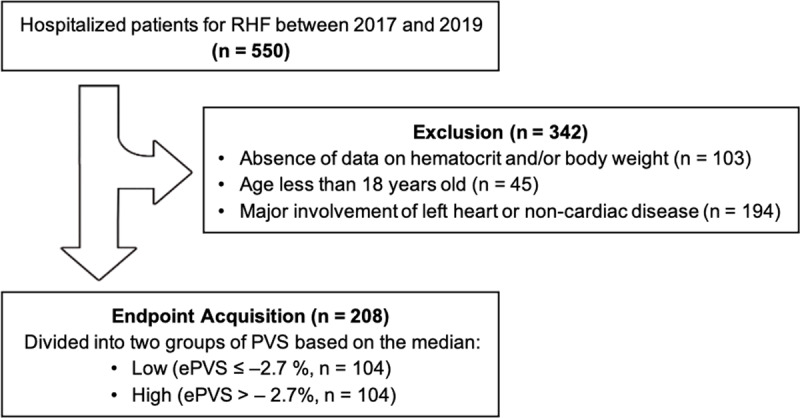
Algorithm Flow of Patient Selection.

Using body weight and hematocrit at admission, ePVS was estimated by subtracting ideal PV (iPV) from actual PV (aPV) based on the Hakim formula: aPV = (1 – hematocrit) × (*a* + [*b* × weight]), with the adjustment factors of *a* = 1,530 in males and 864 in females, and *b* = 41 in males and 47.9 in females; [14] iPV = *c* × weight with the adjustment factors of *c* = 39 in males and 40 in females; [15] ePVS = ([aPV – iPV]/iPV) × 100% [7]. Patients were stratified into low and high groups according to the median value of ePVS at admission (Figure 1) [13]. The Hakim formula was chosen in this study compared to Kaplan formula, which also comprises of body weight hematocrit, because in the previous study in acute left-sided HF population, ePVS calculated with the Hakim formula was the only PV index correlated with the endpoint (all-cause mortality and unplanned hospitalization for worsening HF) [10].

Clinical characteristics between both groups were also compared, including age, gender, etiology of RHF, vital signs, comorbid (anemia, atrial fibrillation, renal insufficiency), laboratory data (serum sodium, potassium, creatinine, urea levels), and medications. The etiology of RHF was classified into pulmonary hypertension (PH) and non-PH (ischemia of the right-sided heart). The subtypes of PH were further divided based on the World Health Organization (WHO) classification [16]. Echocardiography was used to diagnose group 1 PH (pulmonary arterial hypertension [PAH]) and group 2 PH (left-sided heart disease, excluded in this analysis). The diagnosis of group 3 PH (lung diseases) was made by imaging modalities, and that of group 4 (chronic thromboembolic PH [CTEPH]) was established by CT scan. Anemia was defined as hemoglobin concentration of <13 g/dl in males and <12 g/dl in females [17]. Estimated glomerular filtration rate (eGFR) was calculated by the Modification of Diet and Renal Disease formula, and renal insufficiency included those with eGFR <60 mL/min.1.73 m2 [18].

### Statistical Analysis

Categorical data were reported as frequencies (percentages), whereas continuous data were presented as means ± standard deviation or median (interquartile range) depending on the distribution of the variables. To compare the differences in categorical and continuous variables, chi-square test and independent *t*-test or Mann-Whitney U test were accordingly used. Bivariate and multivariate logistic regression models were performed to respectively analyze predictors of events and adjust for potential covariates. Bivariate parameters with a *p*-value <0.10 that can theoretically confound the main association as well as variables previously shown to have prognostic significance [19] were included in the multivariate analysis in which those with a two-sided *p*-value <0.05 were considered statistically significant.

## Results

The baseline characteristics of the 208 RHF patients grouped by ePVS are shown in Table 1. The median of aPV and iPV were respectively 1981 (1662–2329) mL and 2000 (1720–2400) mL, and the mean of ePVS was –4.5 ± 16.4%. Marked heterogenicity on volume distribution of estimated values were demonstrated with diverse PV values, ranging from less than (119; 57%), or equal to 0 (13; 6%) to greater than 0 (76; 37%).

**Table 1:** Baseline Characteristics of Right Heart Failure Patients according to ePVS.


	TOTAL (n = 208)	ESTIMATED PLASMA VOLUME STATUS		*P-VALUE*

		LOW (n = 104)	HIGH (n = 104)	

**Demographic**				

Age (years)	38 (31–50)	38 (31–45)	42 (32–58)	**0.009**

Gender (female, %)	160 (76.9%)	80 (76.9%)	80 (76.9%)	1.00

Weight (kg)	50 (43–60)	57 (48–69)	45 (40–54)	**<0.001**

**Etiology**				

PH				

WHO Group 1 (yes, %)	117 (56.3%)	58 (49.6%)	59 (50.4%)	0.89

WHO Group 3 (yes, %)	28 (13.5%)	10 (9.6%)	18 (17.3%)	0.10

WHO Group 4 (yes, %)	58 (27.9%)	33 (31.7%)	25 (24.0%)	0.22

Non-PH (yes, %)	5 (2.40%)	3 (2.9%)	2 (1.9%)	0.65

**Vital Signs**				

Systolic BP (mmHg)	106 (92–122)	108 (98–122)	101 (89–119)	**0.025**

Diastolic BP (mmHg)	64 (58–77)	69 (60–78)	62 (56–71)	**0.017**

Heart rate (beats/min)	95 (83–108)	96 (86–107)	94 (82–109)	0.68

**Clinical History**				

Anemia (yes, %)	60 (29.0%)	8 (7.7%)	52 (50.5%)	**<0.001**

Atrial fibrillation (yes, %) Renal insufficiency (yes, %)	56 (26.9%) 62 (30.1%)	21 (20.2%) 27 (26.2%)	35 (33.7%) 35 (34.0%)	**0.029** 0.22

**Clinical Chemistry**				

Hemoglobin (g/dL)	13.9 ± 2.9	15.7 ± 2.5	12.1 ± 1.9	**<0.001**

Hematocrit (%)	41.5 (37.1–47.9)	47.9 (42.5–52.8)	37.3 (33.5–40.5)	**<0.001**

Sodium (mmol/L)	135 (131–137)	136 (132–137)	135 (129–138)	0.53

Potassium (mmol/L)	3.8 ± 0.7	3.9 ± 0.7	3.8 ± 0.7	0.14

Creatinine (mg/dL)	1.0 (0.8–1.3)	1.0 (0.8–1.2)	0.9 (0.7–1.4)	0.76

Urea (mg/dL)	40.5 (25.8–70.0)	40.3 (25.8–69.2)	40.5 (26.8–73.0)	0.70

eGFR (mL/min.1.73 m^2^)	81 (51–107)	82 (59–106)	81 (45–107)	0.66

**Medication**				

ACE-I/ARB (yes, %)	144 (69.2%)	64 (61.5%)	80 (76.9%)	**0.016**

Beta-blocker (yes, %)	74 (35.6%)	37 (35.6%)	37 (35.6%)	1.00

Spironolactone (yes, %)	137 (65.9%)	67 (64.4%)	70 (67.3%)	0.66

Furosemide (yes, %)	190 (91.3%)	94 (90.4%)	96 (92.3%)	0.62

Sildenafil (yes, %)	132 (63.5%)	70 (67.3%)	62 (59.6%)	0.25

Digoxin (yes, %)	45 (21.6%)	20 (19.2%)	25 (24.0%)	0.40

Inotrope (yes, %)	51 (24.5%)	23 (22.1%)	28 (26.9%)	0.74

Vasopressor (yes, %)	46 (22.1%)	22 (21.2%)	23 (23.1%)	0.42

**Clinical Outcomes**				

Length of stay (days)	7 (4–11)	8 (5–11)	7 (4–11)	0.53

In-hospital mortality (yes, %)	26 (12.5%)	7 (6.7%)	19 (18.3%)	**0.012**


All p-values were determined using chi-square test for categorical variables and independent t-test or Mann-Whitney U test for continuous variables. Bold represent significant values (<0.05). eGFR, estimated glomerular filtration rate; ACE, angiotensin converting enzyme inhibitor; ARB, angiotensin receptor blocker; BP, blood pressure; PH, pulmonary hypertension; WHO, World Health Organization.

After dividing patients based on the median ePVS, those in the high group (ePVS > –2.7%) were more likely to be older with lower body weight and blood pressure. These patients had not only greater proportions of anemia, atrial fibrillation, and reduced kidney function but also higher use of renin-angiotensin-aldosterone system (RAAS) inhibitors than those in the low group (ePVS ≤ –2.7%). The overall in-hospital mortality was 26 (12.5%), which almost tripled from 7 (6.7%) in the low group to 19 (18.3%) in the high group, and the median time of event was 6 (3–19) days. ePVS significantly predicted study outcome (Table 2) both before and after adjustment for cofounding factors (unadjusted OR: 3.10, 95% CI: 1.24–7.73; adjusted OR: 4.27, 95% CI: 1.32–13.83). Sodium, diuretic, and inotrope use had strong relationship with the impact of ePVS on mortality whereas other clinical covariates were not potent predictors in the study population (Table 2). As linearity test showed that all continuous independent variables were linearly related to the logit of the dependent variable, and every unit decrease of serum sodium concentration was associated with increased risk of death.

**Table 2:** Association between ePVS and In-Hospital Mortality among Right Heart Failure Patients.


	BIVARIATE		MULTIVARIATE	
	
	OR (95% CI)	*p-VALUE*	OR (95% CI)	*p-VALUE*

**Estimated Plasma Volume Status**				

High	3.10 (1.24–7.73)	**0.015**	5.83 (1.62–20.95)	**0.007**

Low				

**Adjusting Factors**				

Age (per 10-year increase)	1.12 (0.85–1.47)	0.460	0.91 (0.59–1.41)	0.684

Gender (male)	1.27 (0.50–3.22)	0.619	1.87 (0.50–6.97)	0.351

Systolic BP (per 10 mmHg increase)	0.90 (0.74–1.09)	0.293	1.16 (0.88–1.53)	0.286

Sodium (per 5 mmol/L increase)	0.49 (0.33–0.74)	**0.001**	0.59 (0.35–1.00)	**0.048**

eGFR (per 10 mL/min.1.73 m^2^ increase)	0.86 (0.77–0.97)	**0.012**	0.96 (0.84–1.10)	0.597

ACE-I/ARB (yes)	0.47 (0.20–1.08)	0.074	0.45 (0.13–1.53)	0.203

Sildenafil (yes)	2.69 (0.97–7.45)	0.058	2.87 (0.78–10.54)	0.112

Furosemide (yes)	0.25 (0.08–0.70)	**0.009**	0.15 (0.03–0.65)	**0.012**

Inotrope (yes)	12.38 (4.91–31.21)	**<0.001**	11.63 (3.59–37.98)	**<0.001**


Bold represent significant values (<0.05). eGFR, estimated glomerular filtration rate; ACE, angiotensin converting enzyme inhibitor; ARB, angiotensin receptor blocker; BP, blood pressure.

## Discussion

### Main Findings

Our analysis confirms that PV expansion estimated from the Hakim formula significantly predicts in-hospital mortality among isolated RHF. However, this relationship may be affected by serum sodium concentration, diuretic, and inotropic agents. These findings suggest that Hakim-derived ePVS could potentially be an easily accessible surrogate for guiding better risk stratification and therapy for patients with RHF.

Congestion, rather than hypoperfusion, is the major reason for HF hospitalization owing to elevated intracardiac filling pressure and neurohormonal changes. Greater degrees of volume overload contribute to multiorgan dysfunction reflecting increased morbidity and mortality [20]. In line with the hypothesis, we revealed that high ePVS had a fivefold increased risk of death, consistent with previous studies on predominant LHF population that a higher ePVS relates to adverse outcomes [8,9,10]. James et al. also reported that volume expansion was a useful prognosticator in patients with PAH because those who died had the highest PV values and were in the terminal stage as presented with advanced RHF [21]. This is also the first study to investigate the threshold for prognostic value of ePVS (> –2.7%) in RHF, and a total of 12.5% patients died within a median of 6 (3–19) days.

Moreover, three clinical covariates were found to closely influence PVS and eventually reflect mortality: sodium, diuretic, and inotrope use. Continuous water retention from inappropriate vasopressin activity due to abnormal regulation of RAAS results in volume overload, hypoosmolality, and consequently dilutional hyponatremia in decompensated HF [22]. Accordingly, our data that serum sodium concentration at admission had a negative relationship with the study outcome supports the results of Forfia et al. that hyponatremia was substantially linked with advanced RHF and reduced survival in PAH patients compared with those with normal serum sodium [23]. This observation suggests that high PV expansion may give rise to low serum sodium concentration, which acts as a marker of disease severity and poor prognosis in RHF.

When treating RHF patients with systemic fluid retention, diuretics are the first-line and mainstay therapy [12]. With an emphasis on loop diuretics, they drive rapid symptomatic relief and improve prognosis by increasing venous capacitance and decreasing cardiac filling pressure [24]. We agreed that furosemide use had a protective effect in RHF; hence, careful titrating of diuretic agents to keep ePVS within the low range might be useful because hemoconcentration through diuresis in decompensated HF was associated with survival, with the consequence of deteriorating renal function [25].

Conversely, inotropes are indicated solely for RHF that presents with inadequate oxygen delivery. Such a condition unfortunately marks end-stage disease where RV dilation shifts the interventricular septum leftward, compresses the LV, and declines effective circulating blood volume [12]. Furthermore, although inotropic agents alter the force and strength of myocardial contractility to restore cardiac output, they have been correlated with short- and long-term mortality in decompensated HF. Dobutamine, the most frequently used inotrope in this study, inherits detrimental effects such as arrhythmogenesis, tachycardia, hypotension, and myocardial ischemia [26]. Likewise, our findings showed that inotrope use was a powerful predictor of outcome and remained inappropriately common in those with high ePVS, and therefore we suggest that this agent should be restricted for patients with contracted PV despite tissue edema.

### Clinical Implications

In this study, RHF is still unexpectedly treated as LHF, not by its etiology. Lack of evidence and clear guidelines available for the predominant RHF syndrome are reflected in its poor treatment options. We found that sildenafil was prescribed less, yet RAAS inhibitors were given routinely, and both had no significant impact on ePVS against mortality in this population. Sildenafil may indirectly lower the backward systemic congestion by reducing RV afterload and improving forward pulmonary circulation. Although sildenafil is a well-established therapy for PAH, it just became nationally accessible in Indonesia in early 2020 [27]. This medication was long replaced by the widely available RAAS inhibitors, the standard drugs for LHF, which are dilemmatic because of their inconsistent benefit in RHF [28]. Preclinical studies on animal models with PAH and RHF have confirmed their use to improve RV remodeling and cardiac output [29], but so far they are not recommended in patients with PAH irrespective of RHF, unless there is an association with LHF, coronary artery disease, or hypertension [30].

Principally, elevated PV is the hallmark of HF syndrome but frequently fails to be recognized because of its insidious onset and limited clinical means. Meanwhile, decongestive therapy is often inadequate, and patients are discharged with residual volume elevation regardless of improving symptoms. In fact, volume optimization is a crucial step of RHF management achieved by determining patients’ volume status on initial examination. ePVS is rapidly available and can be utilized to monitor PV fluctuations in the absence of gross volume overload, which is beneficial for facilitating better therapy and risk stratification. Derived from readily accessible parameters (hematocrit and body weight), this measure is also a lot cheaper considering how accessible hemogram is, and thus it is more desirable, especially in lower- and middle-income countries where poor access to echocardiography and imaging is still profound [31]. Therefore, detection of hemodynamic congestion using this equation is crucial to establish a window for prompt intervention.

Our recommendations on the utility of ePVS-driven RHF management follow the principle that this systemic biomarker essentially estimates intravascular volume overload regardless of clinical congestion. Because impending decompensations could be detected, better risk stratification could be achieved by cautiously treating those with hyponatremia and safely discharging patients without residual subclinical congestion. The Hakim formula could also aid in determining not only when and how much diuretics should be titrated to keep PV low or prevent symptoms in asymptomatic patients but also to whom inotropes should be given to minimize the risk of death. Ultimately, hospitalization could be avoided and mortality could be reduced [7].

### Limitations

Limitations are mostly related to its single-center and retrospective nature. Nevertheless, our study was conducted at the national referral hospital for cardiovascular diseases in which over 200 hospitalized patients with various ethnicity were included in this analysis. Body weight at admission may also not accurately represent dry weight because patients may have already been in the volume overload state. The causes of morbidity in the patients were also not deeply explored in this study. Despite the abovementioned limitations, this study presents new insights into the impact of ePVS on RHF and its management.

## Conclusion

In summary, ePVS is associated with in-hospital mortality among patients with isolated RHF. Given not only wide accessibility of hemogram but also low cost and rapid quantification of ePVS, this simple tool can potentially optimize RHF management. Further multicenter prospective cohorts are warranted to generalize and assess the clinical potential of ePVS in an isolated RHF population.

## Data Accessibility Statement

The data will be available for the readers if requested for further RHF epidemiology research.

## Abbreviations

aPV: actual PV

eGFR: estimated glomerular filtration rate

ePVS: estimated plasma volume status

HF: heart failure

iPV: ideal plasma volume

LHF: left heart failure

LV: left ventricle

LVEF: left ventricle ejection fraction

PAH: pulmonary arterial hypertension

PH: pulmonary hypertension

PV: plasma volume

PVS: plasma volume status

RA: right atrium

RAAS: renin-angiotensin-aldosterone system

RHF: right heart failure

RV: right ventricle

TAPSE: tricuspid annular plane systolic excursion
